# Analysis of the Segregation Distortion of *FcRAN1* Genotypes Based on Whole-Genome Resequencing of Fig (*Ficus carica* L.) Breeding Parents

**DOI:** 10.3389/fpls.2021.647599

**Published:** 2021-08-10

**Authors:** Hidetoshi Ikegami, Kenta Shirasawa, Hiroshi Yakushiji, Shiori Yabe, Masaru Sato, Takeshi Hayashi, Kosuke Tashiro, Hitoshi Nogata

**Affiliations:** ^1^Fukuoka Agriculture and Forestry Research Center Buzen Branch, Yukuhashi, Japan; ^2^Kazusa DNA Research Institute, Kisarazu, Japan; ^3^Institute of Fruit Tree and Tea Science, National Agriculture and Food Research Organization, Higashihiroshima, Japan; ^4^Institute of Crop Science, National Agriculture and Food Research Organization, Tsukuba, Japan; ^5^Faculty of Agriculture, Kyushu University, Fukuoka, Japan

**Keywords:** segregation distortion, male-biased, genome-wide linkage analysis, embryo, gametophyte

## Abstract

The common fig (*Ficus carica* L.) has a gynodioecious breeding system, and its sex phenotype is an important trait for breeding because only female plant fruits are edible. During breeding to select for female plants, we analyzed the *FcRAN1* genotype, which is strongly associated with the sex phenotype. In 12 F_1_ populations derived from 13 cross combinations, the *FcRAN1* genotype segregation ratio was 1:1, whereas the M119-226 × H238-107 hybridization resulted in an extremely male-biased segregation ratio (178:7 = male:female). This finding suggests that the segregation distortion was caused by some genetic factor(s). A whole-genome resequencing of breeding parents (paternal and maternal lines) identified 9,061 high-impact SNPs in the parents. A genome-wide linkage analysis exploring the gene(s) responsible for the distortion revealed 194 high-impact SNPs specific to Caprifig6085 (i.e., seed parent ancestor) and 215 high-impact SNPs specific to H238-107 (i.e., pollen parent) in 201 annotated genes. A comparison between the annotated genes and the genes required for normal embryo or gametophyte development and function identified several candidate genes possibly responsible for the segregation distortion. This is the first report describing segregation distortion in *F. carica*.

## Introduction

During cross breeding, the import frequency of alleles into gametes often varies in the progeny population or a specific gene combination causes sterility, resulting in a segregation ratio that is inconsistent with Mendelian inheritance (i.e., segregation distortion). Segregation distortions have been detected in a wide range of taxa (Burt and Trivers, 2006) and are increasingly recognized as a potentially powerful evolutionary force (Taylor and Ingvarsson, 2003). Segregation distortion is thought to have diverse causes, some of which involve gametophytic lethality, embryonic lethality, or cross-incompatibility (Lashermes et al., 2001; Candela et al., 2011) or are related to (selfish) genetic elements that can enhance their own transmission (Dawkins, 1976; Werren et al., 1988). The selfish genetic factors include Cytoplasmic male sterility (Lewis, 1941), B chromosome (Ostergren, 1945), X chromosome drive (Gershenson, 1928; Sturtevant and Dobzhansky, 1936), Y chromosome drive (Hickey and Craig, 1966; Sweeny and Barr, 1978; Wood and Newton, 1991), t haplotype (Dobrovolskaia-Zavadskaia and Kobozieff, 1927), and transposable factors (McClintock, 1950).

Fig (*Ficus carica* L.) is a dioecious or gynodioecious species, and because only female plant fruits are edible, sex is an important target trait during breeding (Stover et al., 2007; Flaishman et al., 2008). The common fig has the following two sexual types: caprifig type (males) with both male and female flowers and fig type (females) with only female flowers. The progeny resulting from a normal fig type (G/G) × caprifig type (G/A) cross should theoretically be 50% fig and 50% caprifig (fig:caprifig = 1:1) (Storey, 1975).

Mori et al. (2017) identified *FcRAN1* and determined that it is highly correlated with the fig sex phenotype on the basis of several analyses, including a genome-wide association study. Additionally, because of a single nucleotide polymorphism (SNP), its alleles are heterozygous (G/A) in males (caprifig type) and homozygous (G/G) in females (fig type) (Storey, 1975). Moreover, *FcRAN1* is the only gene in the fig genome that carries a polymorphism that perfectly corresponds to the sex phenotype (Mori et al., 2017). In our current fig breeding program, we genotyped the *FcRAN1* alleles of some breeding populations to identify sex phenotypes. We observed that individuals with male type alleles are produced at a high rate only in a specific cross combination (M119-226 × H238-107), suggesting that the *FcRAN1* locus is affected by segregation distortion. Clarifying the cause of this distortion may lead to improved sex control and selection during fig breeding, but it may also provide insights into sex ratio transitions and sex chromosome evolution in fig.

In the present study, we examined the segregation distortion in fig breeding populations and performed preliminary analyses to explore the responsible genetic factors. More precisely, we investigated the distribution of SNPs specific to each pollen and seed parent generating segregation distortion in a whole-chromosome pseudomolecule and considered the biological and evolutionary background related to the occurrence of the segregation distortion.

## Results

**FIGURE 1:**
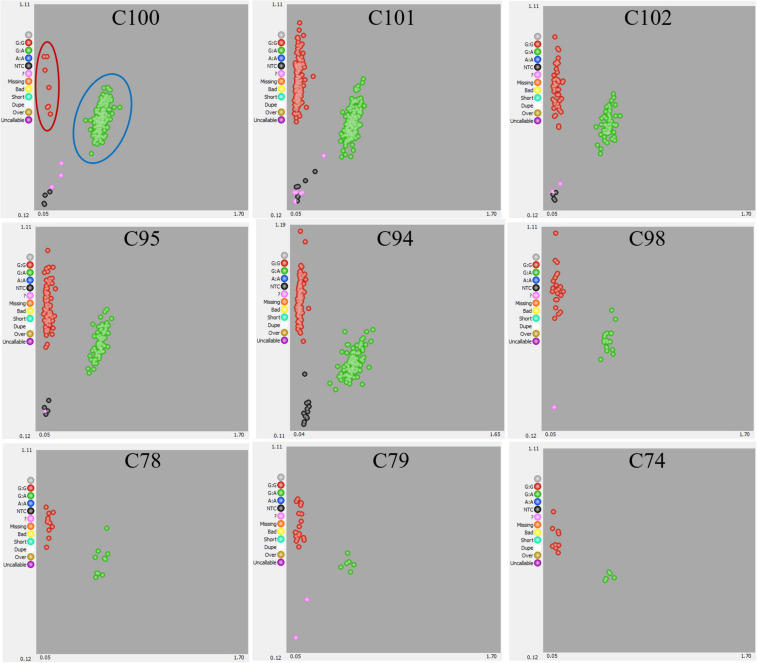
Genotyping results of *FcRAN1* SNP by KASP assay. Genotypic results for 10 progenies derived from 10 cross combination using 7 pollen parent lines. The scatter plot with axes x and y represents allelic discrimination of *FcRAN1* genotypes. The red, green dots represent the A/A homozygous (female fig type), A/G heterozygous (male caprifig type) respectively. NTC, no template controls, or empty.

**FIGURE 2:**
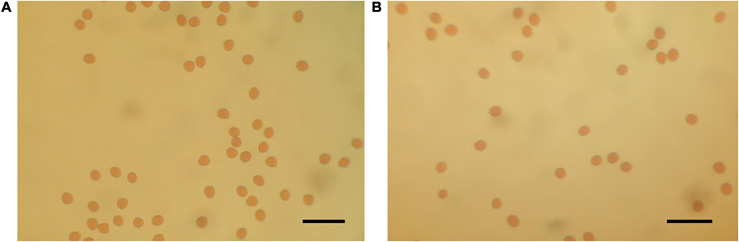
Optical microscope images of stained pollen grains **(A)** H238-107 (distorting line: left) and **(B)** Kibaru (CH13) (non-distorting line: right). Bar = 40 μm.

Crossing experiments involving 10 cross combinations of seven seed parents (M119-226, V180-13, Masui Dauphine, H180-2, HIG5, Toyomitsuhime, and Burjassotte Greece) and six pollen parents (H238-107, M238-1, VD238-83, VS238-53, NG60, and CH-13) generated 1,232 seedlings. The progeny population sizes ranged from 12 to 370 (Table 1). Three cross combinations involving three seed parents (Violette de Dauphine, Violette de Solies, and Royal Vineyard) and two pollen parents (M106-238 and VC-180) from a previous study (Mori et al., 2017) were also analyzed. In 12 of the 13 cross combinations, the segregation ratio of the male (G/A) and female (G/G) genotypes of *FcRAN1* was close to the theoretical segregation ratio of 1:1, ranging from 3:9 to 192:176 (G/A:G/G; χ^2^-test: *p* = 0.014–1.00). In contrast, for the cross combination (C100) involving the seed parent M119-226 and the pollen parent H238-107, the segregation ratio was 178:7 (G/A:G/G; χ^2^-test: *p* < 2.2e-16) (Table 1 and Figure 1). An examination of pollen conditions using an optical microscope revealed that more than 99% of the pollen grains were clearly stained and had a normal appearance in all parental lines, including H238-107 (Figure 2).

**TABLE 1:** F-test for the segregation distortion of the *FcRAN1* genotype in the F_1_ population.

				***FcRAN1* genotype**			
**Progeny ID**	**Seed parent**	**Pollen parent**	**Number of plants**	**Male type (A/G)**	**Female type (G/G)**	**n.d.**	***P* value**
C100	Ml19-226	H238-107	188	178	7	3	2.2E-16
C101	V180-13	M238-1	370	192	176	2	0.404
C102	V180-13	VD238-83	116	58	55	3	0.778
C95	Masui Dauphine	VS238-53	167	83	83	1	1.000
C94	Masui Dauphine	VD238-83	273	138	135	0	0.856
C98	HI80-2	VD238-83	42	19	22	1	0.639
C78	HIG5	NG60	23	10	11	2	0.827
C79	Toyomitsuhime	NG60	26	6	18	2	0.014
C74	Masui Dauphine	CH-13	15	6	9	0	0.439
C205	Burjassotte Greece	NG60	12	3	9	0	0.083
M2017-1	Violette de Dauphine	Ml06-238	71	36	35	0	0.906
M2017-2	Violette de Solies	VC-180	58	20	38	0	0.018
M2017-2	Royal Vineyard	VC-180	62	24	38	0	0.075
Total			1,423	773	636	14	

*The results for lines M2017-1, M2017-2, and M2017-3 are from an earlier study by Mori et al. (2017).*

**FIGURE 3:**
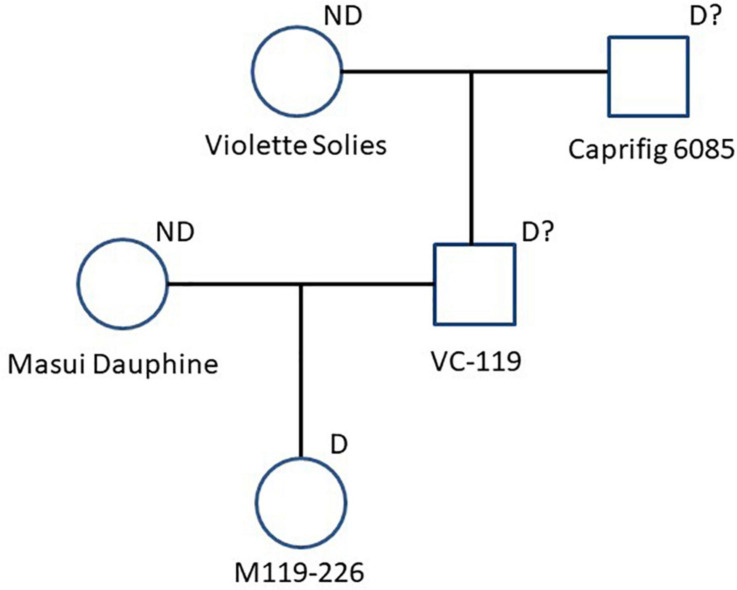
Pedigree chart related to “M119-226.” Seed parent (Masui Dauphine) and paternal grand seed parent (Violette Solies) are marked with “ND” as parents that didn’t cause segregation distortion. “M119-226” and the other genotypes are marked with “D” or “D?” as parents that caused or could cause segregation distortion.

**FIGURE 4:**
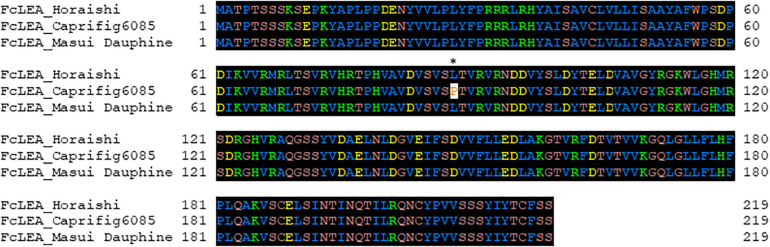
Amino acid sequence alignment of *FcLEA* genes (LC639910). “Horaishi” is the variety of the reference genome. “Caprifig6085” indicates a variety with segregation distortion, and “Masui Dauphine” indicates a representative varietie of the group without segregation distortion. The leucine residue marked with an asterisk (*) in *FcLEA* was mutated to proline to make a non-synonymous substitution. Numbers in left side of sequence indicate the position of the beginning amino acid residue in each protein sequence.

To identify the factor(s) responsible for the segregation distortion, a genome-wide linkage analysis (GWLA) was performed using the whole-genome sequence of each seed and pollen parent. However, the seed parent of C100, M119-226, died after seeds were acquired, preventing it from being analyzed. Therefore, the genome sequence of Caprifig6085, which was an ancestral genotype of M119-226 and may have a genomic region associated with segregation distortion, was used for seed parent analysis as an alternative to M119-226 (Table 1 and Figure 3) (Segregation distortion was not observed in the mating with Masui Dauphine and Violette de Solies as parents). The 4,614,159 SNPs generated by Shirasawa et al. (2021, unpublished data) included 9,061 high-impact SNPs in the seed and pollen parents. The GWLA using the SNP panel indicated that 194 high-impact SNPs were specific to Caprifig6085 as the seed parent and 215 high-impact SNPs were specific to H238-107 as the pollen parent. The functional annotation of these SNPs on the basis of a BLAST analysis produced information for 201 SNPs (98 and 103 SNPs for the seed and pollen parents, respectively) (Supplementary Tables 1, 2). The genes with high-impact SNPs included a *LEA* gene (i.e., encoding a late embryogenesis abundant protein). A sequence analysis revealed that the fig *LEA* gene (designated as *FcLEA1*) is a single-copy gene located on chromosome Fc01a, which also includes the *FcRAN1* locus (Fc01a: 181,985–187,609). Moreover, the polymorphic site in *FcLEA1* corresponds to a non-synonymous substitution that changes a leucine to a proline (Figure 4).

**FIGURE 5:**
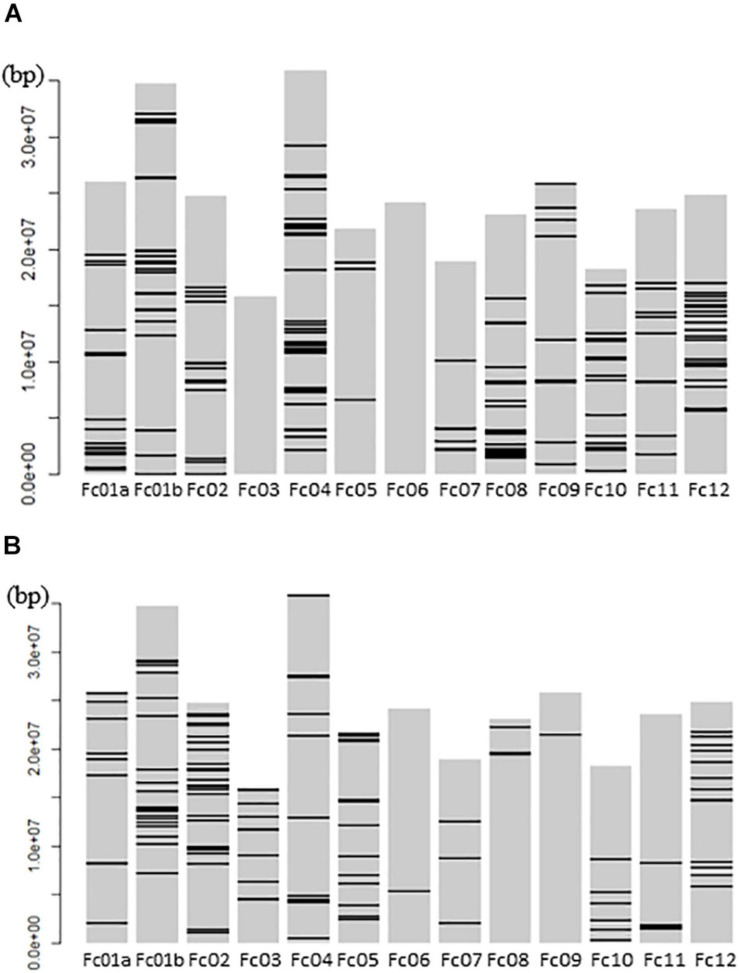
Schematic Diagram showing GWLA SNPs that mapped to the whole chromosome pseudomolecules. **(A)** High impact SNP marker map in pollen parents and **(B)** high impact SNP marker map in seed parents. Horizontal lines on each chromosome indicate the location of SNP markers. The left side number indicate the distance from a telomere point of Fc01a chromosome pseudomolecule.

The mapping of all GWLA SNPs to whole pseudomolecules indicated that high-impact SNPs were widely distributed throughout the genome in the seed and pollen parents, but there were no high-impact SNPs on chromosomes Fc03 and Fc06 of the seed parents (Figure 5).

## Discussion

It is unclear whether the observed segregation distortion is caused by gametophytic lethality, embryonic lethality, or specific genetic events. Accordingly, in this study, a GWLA was performed assuming that the causative factor is located on the chromosomes of either the seed parent (M119-226) or the pollen parent (H238-107) of the population with distorted segregation. Unfortunately, all M119-226 plants died before their genome sequence data could be obtained, making it impossible to further analyze line M119-226. However, there were four ancestral lines of M119-226, namely Caprifig6085, VC-119, Masui Dauphine, and Violette de Dauphine. Both Masui Dauphine and Violette de Dauphine are not associated with segregation distortion (Table 1). Therefore, during the analysis of the seed parents, it was assumed that the Caprifig6085 and VC-119 genomes include a causative factor of segregation distortion. In this study, Caprifig6085 was used as a substitute for M119-226.

Since it is assumed that there are a wide variety of factors that can cause segregation distortion, the number of candidate genes involved is considered to be large corresponding to be the number of factors. For example, in *Arabidopsis thaliana*, 510 *EMBRYO-DEFECTIVE* (*EMB*) genes (Meinke, 2020) and 129 genes associated with female gametophyte deficiency (Pagnussat et al., 2005) have been identified, all of which may contribute to segregation distortion. During the analysis of the seed parents, 98 genes were detected as specific to M119-226. Additionally, we identified several genes similar to the above-mentioned *A. thaliana* genes related to female gametophyte deficiency.

The fertilization of the *A. thaliana* mutant line UNE15, which has a mutation in a gene encoding a LEA protein, is reportedly abnormal because of defective pollen tube guidance (Pagnussat et al., 2005). Accordingly, the female gametophytes of the seed parent M119-226 may be infertile because of a mutation similar to that in UNE15. In addition to the *LEA* gene, other candidate genes may be responsible for segregation distortion, including genes encoding proteins with unknown functions.

If segregation distortion is due to male gametophytic lethality, the causal allele (gene) is likely located on the X chromosomes of the pollen parents because most of the genotyped alleles in the C100 population were the G/A allele derived from the Y chromosome. However, an examination of the pollen used for mating indicated there were no differences in the shapes and appearance of the pollen grains between the pollen parent lines with and without distorted segregation, implying the distortion was at least independent of the pollen physiological state. It remains possible there is a defect in the process after the pollination event (e.g., pollen tube elongation). The TATA element modulatory factor (TMF) (FCA_r2.3chr09: 8,140,218) may affect the segregation distortion. The *A. thaliana* TFIID factor AtTAF6 (i.e., a TATA-binding protein) controls pollen tube growth (Lago et al., 2005). Additionally, TMF binds to the HIV-1 TATA element and inhibits the transcriptional activation by the TATA-binding protein (Garcia et al., 1992).

**FIGURE 6:**
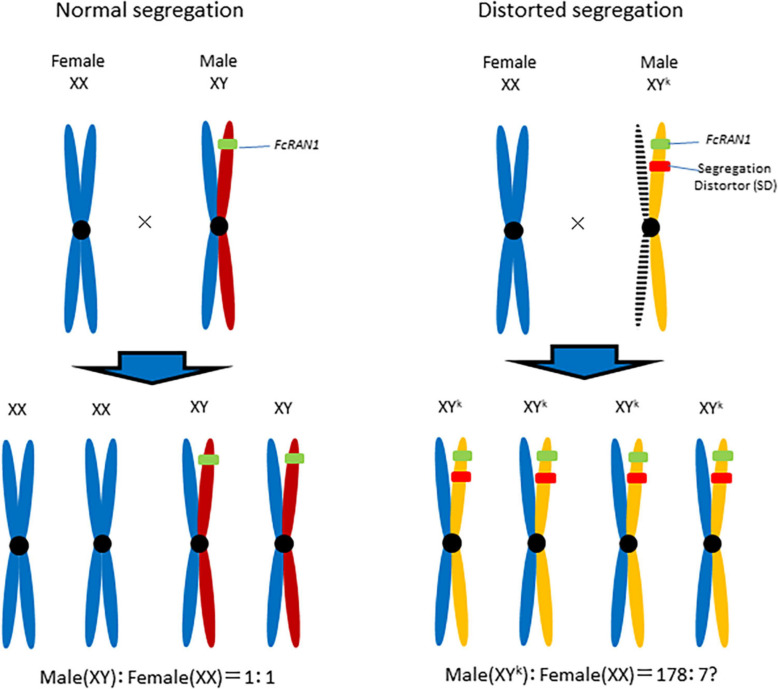
Y chromosome drive model for the role of distorter in Segregation Distortion (SD). Segregation distorter, on the fig Y-chromosome, is expressed during male meiosis to cut a target sequence on the X-chromosome. The shredding of the X-chromosome favors the unaffected Y-carrying gametophyte and results in the production of a male-biased progeny.

Although the current study did not determine whether *FcRAN1* is a true sex-determining gene in *F. carica*, a male-biased *FcRAN1* genotype ratio suggests that Y chromosome drive (Voelker, 1972; Sweeny and Barr, 1978; Wood and Newton, 1991; Presgraves et al., 1997) is important (Figure 6). For a Y chromosome drive system to be functional, the distorter gene and *FcRAN1* (i.e., potential sex-determining gene) should be at different loci on the same Y chromosome (Fc01a) of the pollen parent. Of the nine genes detected on chromosome Fc01a (Supplementary Table 2), the RNase H-encoding gene should be investigated for its potential role in segregation distortion. Simoni et al. (2020) introduced a sex-distorter (I-*Ppo*I) and a CRISPR-based gene drive into mosquito (*Anopheles gambiae*), resulting in stable Y chromosome drive and decreases in the proportion of male individuals in successive generations. Thus, a site-specific DNA double-strand break system involving endonucleases, including I-*Ppo*I, is necessary for a functional Y chromosome drive (Ohle et al., 2016).

The *FcRAN1* KASP marker analysis indicated that some plants in the C100 population had the G/A genotype. There are two possible explanations for this observation. First, the effect of the distorter may have been incomplete. Second, specific events (e.g., chromosomal recombination) may have occurred in which the distorter gene was not activated. Future studies should further characterize the genetic background of the distortion via crossing experiments involving multiple seed parents, including H238-107, and the female C100 progeny. Moreover, because the candidate genes discussed herein were selected on the basis of bioinformatics data, their functions will need to be validated through genetic transformation or genome editing.

The high-impact SNPs in the pollen parents were within 5 Mbp from the end of Fc01a (Figure 5), suggestive of a relationship between the sex-linked region and the segregation distortion. Because the sex-determining gene (i.e., *FcRAN1* or a nearby gene) basically affects the population sex ratio, the distorter gene and *FcRAN1* are likely located close together and behave similarly.

The biological or evolutionary significance of the segregation distortion observed in figs is unclear. However, the occurrence of segregation distortion can change the genetic composition of the species population and may maintain or increase the genetic diversity of the population. In fact, segregation distortion reportedly leads to stable large-scale genomic differences between males and females in hybrid ants (Kulmuni et al., 2010).

Because the *FcRAN1* genotype is strongly correlated with the sex phenotype, the genotypic distortion could influence the evolution of sexual reproduction and sex chromosomes in various ways (Kozielska et al., 2010). Fig X and Y chromosomes are homomorphic, with no significant differences in their molecular structure (Storey, 1975), indicating that fig sex chromosomes are like autosomal chromosomes and are not well differentiated. Therefore, clarifying the segregation distortion of the *FcRAN1* genotype may help elucidate the differentiation of fig sex and autosomal chromosomes.

## Materials and Methods

Seven seed parents (M119-226, V180-13, Masui Dauphine, H180-2, HIG5, Toyomitsuhime, and Burjassotte Greece) and six pollen parents (H238-107, M238-1, VD238-83, VS238-53, NG60, and CH13) were used for hybridizations, which were completed between 1989 and 2018 according to Storey (1975). The obtained seeds were sown in a seedbed and the resulting young seedlings were transplanted to 9-cm pots containing dedicated soil. Leaf samples were collected from the seedlings for KASP and CAPS marker analyses as described below.

### KASP and CAPS Marker Analyses

Of the 1,232 plants resulting from 10 cross combinations, 1,220 (i.e., from the C100, C101, C102, C95, C94, C98, C78, C79, and C74 populations) were included in the *FcRAN1* KASP marker analysis, which was performed according to the manufacturer-recommended protocol for the LGC Genomics high-throughput genotyping system. The KASP Master mix and consumable materials were supplied by LGC Genomics (Middlesex, United Kingdom). A PCR primer pair, *FcRAN1*_f (5′-AGATCCTTAGTTGATGGGGT-3′) and *FcRAN1*_r (5′-CCTCAAACATGTTTAGACTG-3′), was designed based on the sequences flanking the SNP in the probe sequence: GAAGGTTTAAATTAC[A/G]TGTTGCTAATCCTT. Additionally, the 12 plants in the C205 population were genotyped using the CAPS marker as described by Mori et al. (2017). The SNP genotyping data generated from the KASP marker analysis were visualized using the SNPviewer software (LGC, Biosearch Technologies, Beverly, MA, United States).

### Pollen Examination

The pollen of each paternal line was immersed undisturbed in a 600 μl 1% (w/v) iodine solution for 5 min, after which 50 μl of the mixture was added to a microscope slide, covered with a cover glass, and examined using an optical microscope. Pollen grains stained orange or brown were considered to be viable and were counted.

### Whole-Genome Resequencing and Data Processing

Total genomic DNA was extracted from the leaves of eight caprifig-type varieties (H238-107, M238-1, VS238-53, M106-238, CH13, VD238-83, NG-60, and VC-180) and eight fig-type varieties (Masui Dauphine, H180-2, HIG5, Toyomitsuhime, Burjassotte Greece, Violette de Dauphine, Violette de Soloes, and Royal Vineyard) using the DNeasy Plant kit (Qiagen, Valencia, CA, United States). Sequencing libraries were constructed using the genomic DNA as previously described (Shirasawa et al., 2016). Sequences were obtained using the HiSeq 2000 system in the paired-end mode, with a read length of 151 bp. The generated data were processed as described by Shirasawa et al. (2020). High-quality reads were selected by trimming adapters with fastx_clipper (parameter: -a AGATCGGAAGAGC) in the FASTX Toolkit (version 0.0.13)^1^ and deleting low-quality bases with PRINSEQ (version 0.20.4) (Schmieder and Edwards, 2011). Reads were aligned to a common fig reference genome sequence (Shirasawa et al., 2021, unpublished data, the data is available upon request) using Bowtie2 (version 2.2.3) (Langmead and Salzberg, 2012). Sequence variants were detected using the mpileup command in SAMtools (version 0.1.19) (Li et al., 2009). High-confidence heterozygous SNPs were selected using VCFtools (version 0.1.12b) (parameters: –minDP5 –minQ 999 –max-missing 0.75) (Danecek et al., 2011). The SNP effects were categorized using SnpEff (version 3.0) The default parameters of SnpEff were used to analyze variant effects (Cingolani et al., 2012). Genes were predicted and annotated according to previousinformation (Shirasawa et al., 2020).

### Linkage Analysis

The GWLAs of 10 seed parents (M119-226, V180-13, Masui Dauphine, H180-2, HIG5, Toyomitsuhime, Burjassotte Greece, Violette de Dauphine, Violette de Soloes, and Royal Vineyard) and eight pollen parents (H238-107, M238-1, VS238-53, CH13, VD238-83, NG-60, M106-238, and VC-180) were performed using the corresponding genotype datasets derived from the whole-genome resequencing. In each panel, only SNPs with a minor allele frequency >5% were used for the linkage analysis. Case-control association analyses treating the non-distorting type as the control and the distorting type as the case for high-, moderate-, and low-impact SNP sets were conducted to calculate allele frequencies using PLINK 1.90 beta (Purcell et al., 2007). The sex phenotype dataset was generated in a recent study (Mori et al., 2017). The SNPs with an F_A (frequency of this allele in cases) of 1.0 or 0.5 and an F_U (frequency of this allele in controls) of 0.0 were selected as candidate SNPs responsible for the difference between distorting and non-distorting lines.

### Accession Numbers

The *FcLEA* gene sequence has been deposited in DDBJ/EMBL/GenBank under accession number LC639910. Genome sequencing reads of caprifig lines have been deposited in the NCBI Sequence Read Archive under accession numbers DRA011350 and DRA012209.

## Data Availability Statement

The datasets presented in this study can be found in online repositories. The names of the repository/repositories and accession number(s) can be found in the article/Supplementary Material.

## Author Contributions

HI and HY designed the study. HI, KS, HY, SY, TH, and HN completed the analyses. HI, KS, HY, SY, MS, TH, KT, and HN interpreted the data. HI, KS, and HY wrote the manuscript. All authors contributed to the article and approved the submitted version.

## Conflict of Interest

The authors declare that the research was conducted in the absence of any commercial or financial relationships that could be construed as a potential conflict of interest.

## Publisher’s Note

All claims expressed in this article are solely those of the authors and do not necessarily represent those of their affiliated organizations, or those of the publisher, the editors and the reviewers. Any product that may be evaluated in this article, or claim that may be made by its manufacturer, is not guaranteed or endorsed by the publisher.

## Footnotes

1
http://hannonlab.cshl.edu/fastx_toolkit

## Supplementary Material

Supplementary Table 1 “Type” refers to the genetic segregation type: 1| 0 means that the target sample is homozygous for the reference sequence and the other samples are homozygous for the other sequence, whereas 0.5| 0 means the target sample is heterozygous for the reference sequence and the other samples are homozygous for the other sequence.

Supplementary Table 2 “Type” refers to the genetic segregation type: 1| 0 means that the target sample is homozygous for the reference sequence and the other samples are homozygous for the other sequence, whereas 0.5| 0 means the target sample is heterozygous for the reference sequence and the other samples are homozygous for the other sequence.
